# Down-regulation of *OsMYB103L* distinctively alters beta-1,4-glucan polymerization and cellulose microfibers assembly for enhanced biomass enzymatic saccharification in rice

**DOI:** 10.1186/s13068-021-02093-8

**Published:** 2021-12-27

**Authors:** Leiming Wu, Mingliang Zhang, Ran Zhang, Haizhong Yu, Hailang Wang, Jingyang Li, Youmei Wang, Zhen Hu, Yanting Wang, Zi Luo, Lin Li, Lingqiang Wang, Liangcai Peng, Tao Xia

**Affiliations:** 1 Biomass & Bioenergy Research Centre, College of Plant Science & Technology, Huazhong Agricultural University, Wuhan, 430070 China; 2 National Key Laboratory of Crop Genetic Improvement, Huazhong Agricultural University, Wuhan, China; 3 Laboratory of Biomass Engineering & Nanomaterial Application in Automobiles, College of Food Science & Chemical Engineering, Hubei University of Arts & Science, Xiangyang, China; 4 Haikou Experimental Station, Chinese Academy of Tropical Agricultural Sciences, Haikou, 570102 China; 5 State Key Laboratory for Conservation & Utilization of Subtropical Agro-Bioresources, College of Agriculture, Guangxi University, Nanning, China; 6 College of Life Science & Technology, Huazhong Agricultural University, Wuhan, 430070 China

**Keywords:** Cellulose synthesis, Cellulose polymerization, Microfibril assembly, *OsMYBs*, Multi-omics, Biomass saccharification, Fragile culm, Rice

## Abstract

**Background:**

As a major component of plant cell walls, cellulose provides the most abundant biomass resource convertible for biofuels. Since cellulose crystallinity and polymerization have been characterized as two major features accounting for lignocellulose recalcitrance against biomass enzymatic saccharification, genetic engineering of cellulose biosynthesis is increasingly considered as a promising solution in bioenergy crops. Although several transcription factors have been identified to regulate cellulose biosynthesis and plant cell wall formation, much remains unknown about its potential roles for genetic improvement of lignocellulose recalcitrance.

**Results:**

In this study, we identified a novel rice mutant (*Osfc9/myb103*) encoded a R2R3-MYB transcription factor, and meanwhile generated *OsMYB103L*-RNAi-silenced transgenic lines. We determined significantly reduced cellulose levels with other major wall polymers (hemicellulose, lignin) slightly altered in mature rice straws of the *myb103* mutant and RNAi line, compared to their wild type (NPB). Notably, the rice mutant and RNAi line were of significantly reduced cellulose features (crystalline index/CrI, degree of polymerization/DP) and distinct cellulose nanofibers assembly. These alterations consequently improved lignocellulose recalcitrance for significantly enhanced biomass enzymatic saccharification by 10–28% at *p* < 0.01 levels (n = 3) after liquid hot water and chemical (1% H_2_SO_4_, 1% NaOH) pretreatments with mature rice straws. In addition, integrated RNA sequencing with DNA affinity purification sequencing (DAP-seq) analyses revealed that the *OsMYB103L* might specifically mediate cellulose biosynthesis and deposition by regulating Os*CesAs* and other genes associated with microfibril assembly.

**Conclusions:**

This study has demonstrated that down-regulation of *OsMYB103L* could specifically improve cellulose features and cellulose nanofibers assembly to significantly enhance biomass enzymatic saccharification under green-like and mild chemical pretreatments in rice. It has not only indicated a powerful strategy for genetic modification of plant cell walls in bioenergy crops, but also provided insights into transcriptional regulation of cellulose biosynthesis in plants.

**Supplementary Information:**

The online version contains supplementary material available at 10.1186/s13068-021-02093-8.

## Background

Cellulose is the most abundant renewable substance convertible for biofuels and biochemicals [1, 2]. As a complicated chemical process in higher plants, cellulose biosynthesis involves in three major steps: initiation of beta-1,4-glucan chain, chain elongation and microfiber assembly [3–5]. Hence, large numbers of genes have been identified to form cellulose synthase complexes (CSCs) for cellulose biosynthesis [6, 7]. For example, *CesA1*, *CesA3*, and *CesA6*-like *CesAs* are characterized for primary wall cellulose synthesis, whereas *CesA4*, *CesA7*, and *CesA8* are essential isoforms for secondary wall cellulose production in *Arabidopsis* [8, 9]. However, despite other genes have been also identified to associate with cellulose synthesis and assembly in plants [6, 10], much remains unknown about their regulation mechanisms.

Over the past years, various transcription factors (TFs) families have been characterized for dynamic regulations of secondary cell wall biosynthesis in plants such as VNDs (VASCULAR-RELATED NAC-DOMAIN), NSTs (NAC SECONDARY WALL THICKENING PROMOTING FACTOR1), NACs (NAM, ATAF1,2, and CUC2) and MYBs (v-myb avian myeloblastosis viral oncogene homolog) [11, 12]. Among the TFs examined, MYBs are identified with specific expressions in plant tissues, and their major targets are secondary cell wall-related genes or TFs for secondary wall thickening such as *MYB46*, *MYB83*, *MYB58*, *MYB63* [13, 14]. Importantly, *MYB103* and *OsMYB103L* have been examined as an R2R3-MYB transcriptional factor for regulating secondary cell wall synthesis in *Arabidopsis* and rice, respectively [15, 16]. In addition, *OsMYB103L* plays an important role in GA-regulating secondary cell wall synthesis in rice [17]. Although *OsMYB103L* has been reported with potential binding with *OsCesAs* genes of secondary cell wall [17], it has not been reported about specific regulation of any TFs for cellulose biosynthesis in rice, particularly to underlie impacts on cellulose feature and microfibrils assembly in plant cell walls.

Cellulose is composed of the beta-1,4-glucan chains that form microfibrils [4]. As a major load-bearing component of plant cell walls, cellulose is embedded with the matrix of hemicellulose and lignin for complicated wall-networks, which not only affects plant biochemical metabolism and mechanic strength, but also determines lignocellulose recalcitrance against biomass enzymatic saccharification [18–20]. Particularly, cellulose crystallinity and polymerization have been characterized as the major lignocellulose recalcitrant factors, which are accountable by measuring crystalline index (CrI) and degree of polymerization (DP) values of cellulose substrates [21–24]. More recently, it has been stated that either *OsCesA* site-mutation or over-production of lignocellulose-degradation enzymes could significantly reduce cellulose CrI and DP values for much enhanced biomass enzymatic saccharification [25–27]. Hence, it becomes interesting to find out a genetic approach for regulating cellulose biosynthesis towards lignocellulose recalcitrance improved in plants.

Rice is a major food crop all over the world and provides large amounts of lignocellulose-based straw for biofuel production [26, 28, 29]. In this study, we identified a novel rice mutant with deletion in the binding motif of OsMYB103L, and then generated RNAi-silenced *OsMYB103L* transgenic lines to explore *OsMYB103L* regulation roles in cellulose biosynthesis and plant cell wall formation. Using both genetic mutant and transgenic lines, we determined specifically reduced cellulose levels in plant cell walls with little alteration of other major wall polymer (hemicellulose, lignin) contents in rice mature straws. Furthermore, this study detected significantly reduced cellulose CrI and DP values with distinct nanofibers assembly, leading to remarkably enhanced biomass enzymatic saccharification examined in the mutant and RNAi transgenic lines. By performing multi-omics analyses, RNA-seq and DAP-seq assays along with gene co-expression profiling, this work found out that the *OsMYB103L* may predominately regulate cellulose biosynthesis in rice, providing a powerful strategy for genetic modification of plant cell walls with little impact on plant growth and biomass yield in bioenergy crops.

## Results

### Identification of novel rice *OsMYB103L* mutant and RNAi lines

**Fig. 1:**
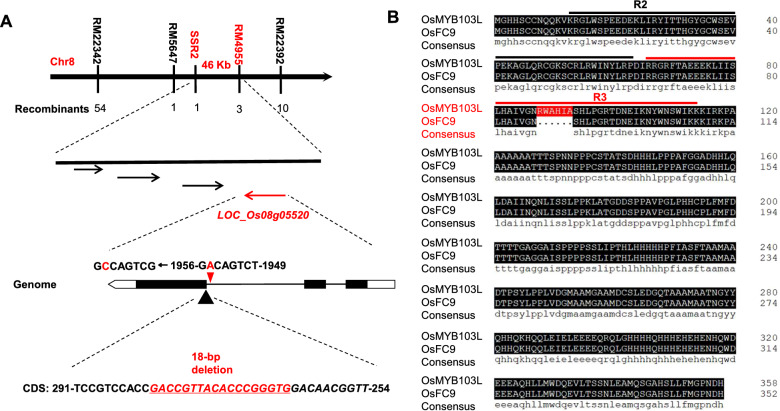
A map-based approach for cloning the *myb103*/*Osfc9* gene. **A** The *Osfc9* gene mapped to the region between markers SSR2 and RM4955 in a 46-kb region on the chromosome 8. The candidate gene of *LOC_Os08g05520* identified with a single-base mutation in the end of the second intron for the 18-bp deletion in CDS sequence. **B** Six amino acid deletion in the R3 binding domain of the mutant

**Fig. 2:**
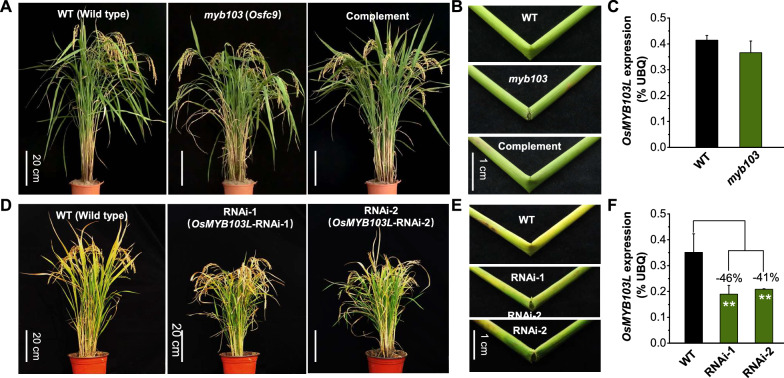
Phenotype observation of *myb103* mutant and *OsMYB103L*-RNAi transgenic lines. **A** Images of WT (NPB, wild type), *myb103* (*Osfc9* mutant) and its complementary line. **B** Stem brittleness of WT, *myb103* and its complementary line. **C** Gene expression analysis of *OsMYB103L* between WT and *myb103*. **D** Images of WT and two *OsMYB103L-*RNAi-transgenic lines (RNAi-1and RNAi-2). **E** Stem brittleness of WT and two *OsMYB103L* RNAi transgenic lines (RNAi-1 and RNAi-2). **F** Gene expression analysis of *OsMYB103L* in WT*,* and two *OsMYB103L* RNAi transgenic lines (RNAi-1 and RNAi-2). **As significant differences of two *OsMYB103L-*RNAi transgenic plants compared with WT at *p* < 0.01 (*n* = 3) with decreased ( −) percentage, respectively

Using our previously established T-DNA mutagenesis pool of the rice variety (*Nipponbare*), this study screened out a fragile-culm mutant, termed *fragile culm 9* (*Osfc9*). By means of total 1050 F_2_ mutant plants generated from a cross between *Osfc9* and an *indica* cultivar MH63, a classic map-based genetic approach was applied to clone the *Osfc9* gene (Fig. 1). The *Osfc9* gene was preliminarily identified between RM22342 and RM22392 on chromosome 8 by using 145 polymorphic SSR markers (Fig. 1A). We further used other three known SSR markers (RM5647, RM22358, RM4955), and five designed primers SSR1-SSR5 (Additional file 2: Table S1) to map the gene between SSR2 and RM4955 in a 46-kb genomic region, which contained four open reading frames. Among the CDS (Coding DNA Sequence) of all candidate genes, only one gene was identified with 18-base deletion in *LOC_Os08g05520*, a R2R3-MYB like transcriptional factor termed as *OsMYB103L*. We then identified a single-base mutation from A (adenine) to C (cytosine) in the end of the second intron, which resulted in an error in the recognition of intron splicing for the deletion of 18-base in CDS of the *Osfc9/myb103* mutant, and the motif analysis indicated 6-amino acid deletion in the R3 binding domain (Fig. 1B). However, further analysis showed that the deletion of 18-base in CDS did not significantly alter the transcript level of *LOC_ Os08g05520* (Fig. 2C), suggesting that missense or termination mutation of *OsMYB103L* should not occur in the mutant.

Meanwhile, this study observed that the *myb103* mutant had a normal plant growth with similar grain yield to the wild type (WT) (Fig. 2A; Additional file 1: Fig. S1D), but it was of obvious fragile-culm phenotype and remarkably reduced plant height which should be the major cause accounting for significantly decreased lodging index examined in the mutant (Fig. 2B; Additional file 1: Fig. S1B, C). To confirm the fragile-culm phenotype, we performed genetic complemental experiment by expressing a 2851-bp full genomic sequence of *LOC_Os08g05520* driven with its upstream 2 kb sequence as promoter in the *myb103* mutant. Notably, the fragile-culm phenotype of the mutant was almost completely restored in the complementation line (Fig. 2A, B).

Furthermore, this study generated *OsMYB103L* RNAi-silencing transgenic lines and examined two independent lines with significantly reduced *OsMYB103L* expression levels by 46% and 41%, compared to WT (Fig. 2F). As a further comparison with the *myb103* mutant, two RNAi lines showed a highly similarity in the fragile-culm phenotype and other major traits examined (Fig. 2D, E; Additional file 1: Fig. S1), suggesting that both mutant and transgenic lines could be applicable to investigate *OsMYB103L* function in rice.

### Reduced cellulose production in *myb103* mutant and RNAi line

**Fig. 3:**
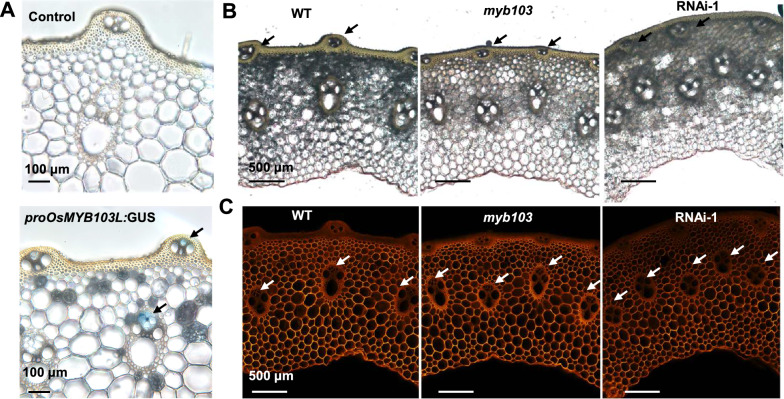
Morphological observation of vascular bundles in stem tissues of *myb103* mutant and RNAi transgenic line. **A** Examination of GUS activity by expressing pro*OsMYB103L*:GUS. **B** View of vascular bundles development and distribution; the stem slices obtained with 100 μm thickness, and imaged using a Nikon Eclipse Ni-U microscope under bright field. **C** S4B cellulose staining; the stem slices obtained with 100 μm thickness, stained with S4B (Direct Red 23, Sigma), and imaged using a Nikon Eclipse Ni-U microscope under 530–560 nm excitation and 620–660 nm emission

As the *myb103* mutant and RNAi transgenic lines were of fragile-culm phenotypes, this study examined their cell wall structures and major wall polymer contents. Using the *β-glucuronidase* (GUS) reporter gene driven by *OsMYB103L* putative promoter, we first observed *OsMYB103L* special expression in vascular bundles (Fig. 3A)*.* Then, we found obviously altered vascular bundles distribution in stem tissues of the *myb103* mutant and RNAi transgenic line, compared to the WT (Fig. 3B, C). For instance, both *myb103* mutant and RNAi line showed an increase in the number of vascular bundles, but had a reduction in the area of single vascular bundle (Fig. 3C). In addition, the distribution of vascular bundles in the epidermis was altered by moving into inside cells in the mutant and transgenic line (Fig. 3B). The alteration of vascular bundles distribution in stem tissues may be one factor accounting for their reduced mechanical strength and culm brittleness.

**Fig. 4:**
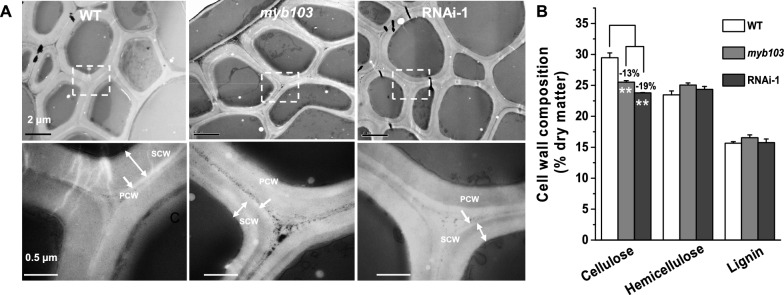
Measurements of cell wall thickness and cell wall composition in *myb103* mutant and RNAi transgenic line. **A** Observation of cell wall thickness by transmission electron microscopy. **B** Cell wall composition analysis. **As significant differences between *myb103* and RNAi-1 with WT at *p* < 0.01 (*n* = 3). SCW, secondary cell wall. *PCW* primary cell wall

Under transmission electron microscopy (TEM), we observed significantly reduced cell wall thickness in *myb103* mutant and RNAi-1 transgenic line at *p* < 0.05 and 0.01 level, compared to the WT (Fig. 4A, Additional file 1: Fig. S2). Accordingly, this study detected significantly reduced cellulose levels by 13% and 19% in mature straws of the *myb103* mutant and RNAi-1 transgenic line, respectively (Fig. 4B), consistent with the Pontamine fast scarlet 4B (S4B) staining of cellulose that showed relatively weaker intensity in the stem tissues of both mutant and transgenic line than that of the WT examined (Fig. 3C). By comparison, other two major wall polymers (hemicellulose, lignin) were not significantly altered in both mutant and transgenic line at *p* > 0.05, compared to WT (Fig. 4B), which was supported by observation of the FTIR spectroscopy profiling that did not show much difference in the characteristic peaks for interlinkages among hemicellulose and lignin such as at 1727, 1605, 1511, 1051, and 754 cm^−1^ in all samples examined [30, 31] (Additional file 1: Fig. S3). Hence, the results suggested that down-regulation of *OsMYB103L* may mainly lead to reduced cellulose biosynthesis for relatively thinner cell walls in both *myb103* mutant and RNAi-1 transgenic line.

### Altered cellulose features and microfibril assembly in *myb103* mutant and RNAi line

**Fig. 5:**
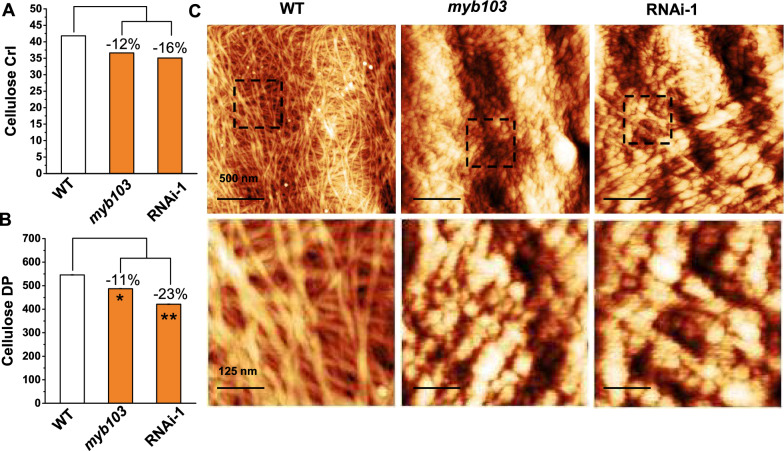
Characterization of cellulose features and microfibril assembly in *myb103* mutant and RNAi line. **A** Cellulose CrI. **B** Cellulose DP. **C** Cellulose nano-microfibrils observed under atomic force microscopy. * and **as significant differences between *myb103* and RNAi-1 with WT at *p* < 0.05 and 0.01 (*n* = 3) with decreased ( −) percentage, respectively

With respect to the *myb103* mutant and RNAi transgenic line that were of specifically reduced cellulose deposition, this study detected two major cellulose features (CrI, DP) in the mature rice straws (Fig. 5). Compared to the WT, the *myb103* mutant and RNAi transgenic line, respectively, showed the decreased cellulose CrI values by 12 and 16% (Fig. 5A), whereas their cellulose DP values were significantly reduced by 11 and 23% at *p* < 0.05 and 0.01 level (Fig. 5B). Furthermore, under atomic force microscopy (AFM), we observed cellulose microfibers assembly in parenchyma-type secondary cell walls of rice stem tissue (Fig. 5C). As a comparison, the WT exhibited a normal cellulose microfibrils orientation in a common direction as a poly-lamellate structure, while both *myb103* mutant and RNAi transgenic line showed a fibril-broken and disordered pattern with a rough surface for cellulose nanofibers accumulation, consistent with the previous findings that low-DP cellulose deposition leads to cellulose nanofibers assembly [25, 32]. Hence, the results suggested that down-regulations of *OsMYB103L* should affect cellulose biosynthesis for significantly reduced cellulose level and features and altered cellulose microfibril assembly.

### Enhanced biomass enzymatic saccharification in *myb103* mutant and RNAi line

**Fig. 6:**
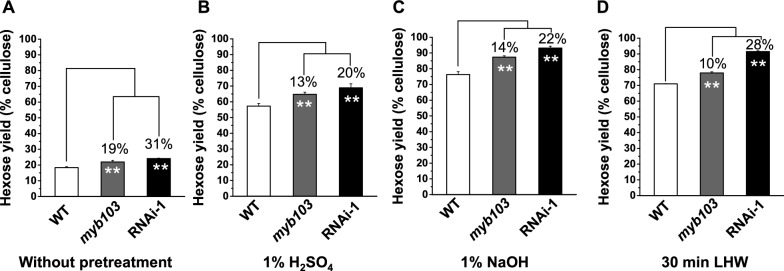
Biomass enzymatic saccharification of *myb103* mutant and RNAi line under different pretreatments. **A** Hexoses yields released from direct enzymatic hydrolysis of raw material (without pretreatment). **B** Hexoses yields released after 1% H_2_SO_4_ pretreatment. **C** Hexoses yields released after 1% NaOH pretreatment. **D** Hexoses yields released under lipid hot water pretreatment (LHW) (30 min). **As significant differences between *myb103* and RNAi-1 with WT at *p* < 0.01, (*n* = 3)

Because cellulose features distinctively affect biomass enzymatic saccharification, this study measured hexose yields (% cellulose) released from enzymatic hydrolysis of pretreated rice straws in the *myb103* mutant and RNAi transgenic line (Fig. 6). Without any pretreatment, both *myb103* mutant and RNAi transgenic line showed significantly higher hexose yields released from enzymatic hydrolysis of raw materials than those of the WT at *p* < 0.01 level with increased rates of 19% and 31% (Fig. 6A). Using mild chemical (1% H_2_SO_4_, 1% NaOH) and liquid hot water pretreatments as previously established [24, 26, 33], we further examined significantly enhanced hexose yields by 10%-28% released from enzymatic hydrolysis of pretreated straw residues in the mutant and transgenic line, compared to the WT (Fig. 6B–D), indicating a consistently enhanced biomass enzymatic saccharification in the *myb103* mutant and *OsMYB103L*-RNAi transgenic line under relatively cost-effective pretreatments.

### Biochemical identification of the OsMYB103L motif for regulating *OsCesAs* expression

**Fig. 7:**
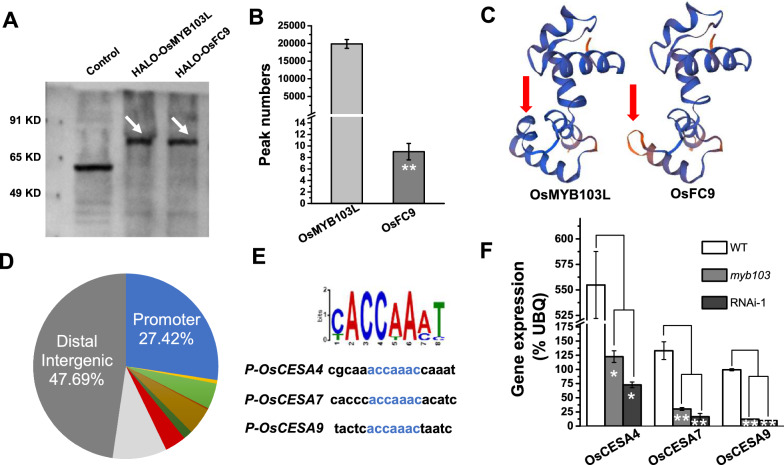
Biochemical identification of OsMYB103L binding sites. **A** Protein expression of HALO-tagged OsMYB103L and OsFC9 in vitro. **B** Peak analysis of OsMYB103L and OsFC9 by DAP-seq. **C** Protein structure prediction between OsMYB103L and OsFC9. **D** Distribution of combined peaks of OsMYB103L by DAP-seq. **E** Motif analysis of combined peaks of OsMYB103L with the most significant *E*-value. **F** Gene expression analysis of *OsCESA4*, *OsCESA7* and *OsCESA9* in WT, *myb103* and RNAi-1. * and **as significant differences between *myb103* and RNAi-1 with WT at *p* < 0.05 and 0.01 (*n* = 3), respectively

To test *OsMYB103L* regulation on cellulose biosynthesis, we performed DAP-seq analysis by using a high-throughput TF binding site discovery method [34, 35]. As a result, the characteristic proteins of OsMYB103L and mutated OsFC9 in vitro were detected by means of wheat germ system (Fig. 7A). Further DAP-seq analysis identified nearly 20,000 peaks across the whole genome of OsMYB103L, whereas less than 10 peaks were found in the mutated OsFC9 protein (Fig. 7B), suggesting that the *myb103* mutation should not affect its protein production, but may result in the significant loss of binding ability with down-stream DNA sites. As the single-base mutation caused 6 amino acid deletion in R3 binding motif of the *myb103*, we predicted the three-dimensional structure of the *myb103* using the SWISS-MODEL (https://swissmodel.expasy.org/) [36]. Compared to the standard OsMYB103L protein structure, the mutated OsFC9 protein was of a lost α-helix region (Fig. 7C). Since the α-helix region plays an important role for DNA binding [37], the results suggested that six amino acids deletion in the R3 motif of OsMYB103L protein should significantly affect down-stream gene expression.

Among the 20,000 peaks examined, this study attempted to identify binding motif and potential target genes, and about 27.42% of the OsMYB103L-binding sites were found in the promoter region (Fig. 7D). From promoter binding sites, the motif NACCNANN showed the most significance (E-value 2.2e-1710), and the motif ACCAAAC (SMRE3) was detectable in the protomers of *OsCesA4*, *OsCesA7* and *OsCesA9* (Fig. 7E), which were also examined with enriched peaks from DAP-seq analysis (Additional file 1: Fig. S4). In addition, we observed *OsMYB103L* localization in nucleus from transient transformation experiment, and detected its transactivation (Additional file 1: Fig. S5). Taken together, this study demonstrated that the *OsMYB103L* could act as an active transcription factor for regulating genes involved in cellulose biosynthesis.

### Genome-wide analysis of *OsMYB103L* regulation on cellulose deposition

**Fig. 8:**
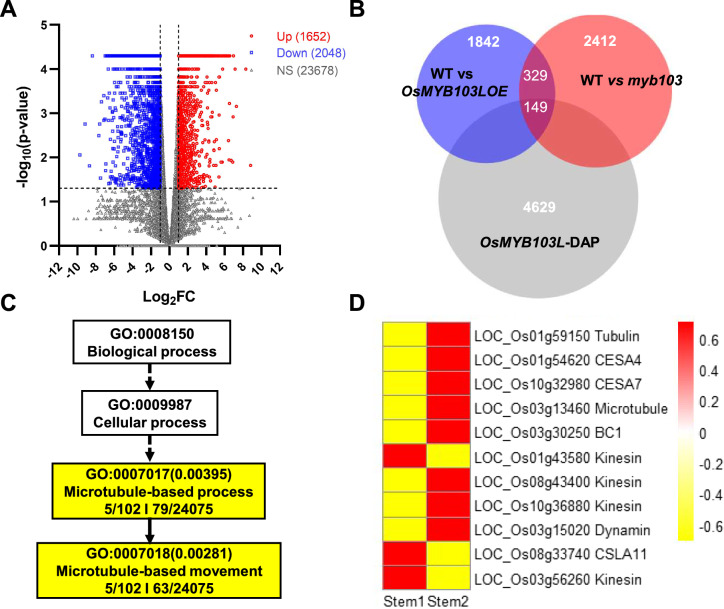
Multi-omics analysis of *OsMYB103L* regulation on cellulose synthesis and assembly. **A** Volcano analysis of significantly expressed genes of *myb103* compared with WT by RNA-seq. **B** The number of genes overlapped by significantly expressed genes in *myb103* mutant and *OsMYB103L*-overexpression plant, and targeted genes of OsMYB103L by DAP-seq. **C** The most significant terms of overlapped genes by GO analysis. **D** Co-expression analysis of 11 overlapped genes in two different stem tissues. Stem 1, stem at 5 days before heading. Stem 2, stem at heading stage

To understand how *OsMYB103L* regulation consequently affects cellulose microfibril assembly, this study performed transcriptome analysis of *myb103* mutant (Fig. 8). As a result, total 1652 genes were significantly up-regulated, whereas 2048 genes were significantly down-regulated with slightly altered expressions of 23,678 genes, compared to the WT (Fig. 8A, Additional file 4: Table S3). Integrating the differentially expressed genes (DEGs) of the *myb103* mutant with ones of the *OsMYB103L*-overexpressed transgenic line as previously reported, total 478 overlapped genes were identified (Fig. 8B). Using DAP-seq analysis, we further identified 149 overlapped genes that were significantly regulated (Additional file 5: Table S4). Based on the AgriGO v2.0 analysis of 149 genes [38], two GO terms were sorted out with significantly enriched such as microtubule-based process and microtubule-based movement (Fig. 8C), which has been characterized to involve in cellulose synthesis and deposition [4].

By means of the microarray datasets of 149 genes from CREP, this study finally screened 11 genes, which were highly expressed in the secondary cell walls of rice stem tissues (Fig. 8D). Among the 11 genes, we identified 3 genes directly participated to catalyze cellulose biosynthesis such as *OsCESA4* (*LOC_Os01g54620*), *OsCESA7* (*LOC_Os10g32980*) and *CSLA11* (*LOC_Os08g33740*), whereas other 8 genes (*LOC_Os03g30250*, *LOC_Os10g36880*, *LOC_Os03g56260*, *LOC_Os08g43400*, *LOC_Os01g43580*, *LOC_Os01g59150*, *LOC_Os03g13460*, *LOC_Os03g50520*) should involve in in cellulose deposition and microfibril assembly (Table 1). Notably, those 11 genes were significantly down-graded in the *myb103* mutant, but 8 of 11 genes (*LOC_Os01g54620*, *LOC_Os10g32980*, *LOC_Os03g30250*, *LOC_Os10g36880*, *LOC_Os01g43580*, *LOC_Os01g59150*, *LOC_Os03g13460*, *LOC_Os03g50520*) were up-graded in the *OsMYB103L*-overexpressed line, which thus interprets why the *myb103* mutant was of reduced cellulose level and features (DP, CrI) for length-reduced cellulose nanofibers accumulation as observed above.

**Table 1:** Candidate genes regulated by *OsMYB103L* for cellulose synthesis and assembly

Biological processes	Genes	Putative function	log_2_(fold_change) in *myb103* mutant vs WT	log_2_(fold_change) in *OsMYB103L* overexpression line vs WT
Cellulose synthesis	*LOC_Os01g54620*	CESA4—cellulose synthase, expressed	− 1.62 down	3.59 up
	*LOC_Os10g32980*	CESA7—cellulose synthase, expressed	− 2.01 down	1.70 up
	*LOC_Os08g33740*	CSLA11—cellulose synthase-like family A, expressed	− 2.55 down	− 4.25 down
Cellulose deposition	*LOC_Os03g30250*	BC1, COBRA-like protein precursor, putative, expressed	− 2.49 down	2.39 up
	*LOC_Os10g36880*	Kinesin motor domain-containing protein, putative, expressed	− 1.67 down	1.54 up
	*LOC_Os03g56260*	Kinesin motor domain-containing protein, putative, expressed	− 1.56 down	− 2.74 down
	*LOC_Os08g43400*	Kinesin motor domain-containing protein, expressed	− 1.30 down	− 2.64 down
	*LOC_Os01g43580*	Kinesin motor domain-containing protein, putative, expressed	− 1.05 down	1.46 up
	*LOC_Os01g59150*	Tubulin/FtsZ domain-containing protein, putative, expressed	− 1.42 down	3.36 up
	*LOC_Os03g13460*	Microtubule-associated protein, putative, expressed	− 1.34 down	2.38 up
	*LOC_Os03g50520*	Dynamin family protein, putative, expressed	− 1.18 down	1.44 up

## Discussion

In this study, we have identified a novel allele mutant (*Osfc9/myb103*), a single-base mutation in the end of the second intron of *OsMYB103L* gene. Because of an error in recognition of intron splicing, the single-base mutation results in the deletion of 18-base in CDS sequence without significant alteration of *OsMYB103L* expression, indicating no missense or termination mutation in CDS sequence (Fig. 1; Fig. 2C). However, this mutation causes 6-amino acid deletion in the R3 binding domain of *OsMYB103L* and significantly alters the three-dimensional structure of OsMYB103L protein, which should be the major factor affecting recognition of down-stream gene sites (Fig. 1B; Fig. 7). In the previous study, *OsMYB103L* was identified from a *culm easily fragile 1* (*cef1*) mutant, and its mutation occurred in the first exon of the CDS sequence with a 13-bp deletion, resulting in a frame-shift and premature translational product of 56 amino acids [17]. Despite of different mutation sites in *OsMYB103L*, both *Osfc9* and *cef1* mutants exhibited significantly decreased stem strength (breaking force) in rice. However, unlike other brittle-culm mutants as previously identified in rice [39, 40], the *Osfc9* mutant has showed relatively slight effect on plant growth with similar agronomic traits to the WT (Fig. 2; Additional file 1: Fig. S1). Hence, the novel allele mutation in R3 domain of *OsMYB103L* provides a genetic strategy for plant cell wall modification in bioenergy crops.

In plants, hundreds of TFs have been identified to involve in plant growth and development, and the NACs, VNDs, NSTs and MYBs are particularly characterized for dynamic regulations of plant cell wall biosynthesis [11, 12]. However, little is yet reported about TF special regulation for cellulose biosynthesis. Although *OsMYB103L* has been examined as the R2R3-MYB transcriptional factor regulating impacting cell wall synthesis and enabled to bind with the promoters of *CesAs* genes [16, 17], it remains to explore if it is specific for regulating cellulose biosynthesis and how cellulose assembly is consequently mediated. As the down-regulation of *OsMYB103L* could predominately lead to remarkable reductions of cellulose levels and features without significant impacts on other two major wall polymers (hemicellulose, lignin) in the *myb103* mutant and RNAi transgenic line (Fig. 4B, Fig. 5A, B), this study has thus provided solid evidences about the *OsMYB103L* special regulation on cellulose biosynthesis. Notably, due to a reduced DP of beta-1,4-glucans, this work has unexpectedly observed distinct cellulose nanofibers assembly in the *myb103* mutant and RNAi transgenic line (Fig. 5), which should further confirm the finding of *OsMYB103L* specific regulation on cellulose biosynthesis in rice.

Since multi-omics analyses have become a powerful tool for gene functional study [41], combined with transcriptome assay and DAP-seq in the *myb103* mutant and *MYB103L*-overexpressed transgenic line, this study has sorted out several well-known genes related to cellulose deposition and assembly in plant cell walls (Table 1). For instances, the *BC1* gene encoded a COBRA-like protein has been proved to control cellulose deposition of secondary cell walls [42], whereas other genes are characterized to mediate beta-1,4-glucans orientation and assembly such as kinesin motor domain, dynamin-related proteins and microtubule-associated protein [43, 44]. In addition, we analyzed the cis-acting elements in the promoters of these 11 candidate genes, and found most of these genes contained the reported cis-acting elements related to cell wall synthesis regulated by *OsMYBs* such as AC-II and SMRE1 (Additional file 6: Table S5). Therefore, this study has further revealed that the *OsMYB103L* could not only regulate cellulose biosynthesis, but also consequently mediate cellulose microfibril assembly, interpreting why the down-regulation of *OsMYB103L* could lead to a broken and disordered cellulose microfibers assembly in the *myb103* and RNAi transgenic line as described above (Fig. 5B).

Cellulose is an important loading-bearing skeletal component to maintain plant mechanical strength, and it also provides the most abundant biomass resource convertible for biofuels and biomaterials. However, the natural lignocellulose recalcitrance basically determines a costly biomass enzymatic saccharification, which is unacceptable for biofuel production at large scale [1, 20]. To reduce recalcitrance, genetic modification of plant cell walls has been considered as a promising solution [39, 45]. Because cellulose features (CrI, DP) are the major factors of lignocellulose recalcitrance [21–24], the *myb103* and *OsMYB103L*-RNAi transgenic line that are of much reduced cellulose CrI and DP values in mature straws (Fig. 5), should enable to provide recalcitrance-improved lignocellulose substrates for significantly enhanced biomass enzymatic saccharification examined in this study (Fig. 6). In addition, due to the length-reduced cellulose nanofibers accumulation, the *myb103* mutant and RNAi line should be applicable to generate high-value and low-cost nanomaterials and nanocarbons. Hence, genetic engineering of transcriptional factors should offer a potential approach for plant cell wall modification in bioenergy crop breeding.

## Conclusions

Using a novel rice *Osfc9/myb103* mutant and *OsMYB103L*-RNAi transgenic line, this study examined that down-regulations of *OsMYB103L* could significantly reduce cellulose crystallinity and polymerization without significant impacts in other major wall polymers (hemicellulose, lignin), which thereby resulted in distinct cellulose nanofiber assembly in plant cell walls. These consequently improved lignocellulose recalcitrance for much enhanced biomass enzymatic saccharification under green-like and cost-effective chemical pretreatments. By integrating RNA-seq analyses with DAP-seq assays, this work attempted to demonstrate that *OsMYB103L* may act as an active transcription factor to specifically mediate cellulose biosynthesis and deposition by regulating *OsCesAs* genes and other genes associated with microfibril orientation and assembly. Hence, this study has provided a powerful strategy for genetic modification of plant cell walls in bioenergy crops.

## Methods

### Selection of rice genetic mutant (*Osfc9*/*myb103*)

Rice (*Oryza sativa*) *fragile culm 9* (*Osfc9*) mutant, termed as *myb103*, was selected from a T-DNA mutagenesis pool of the *japonica* variety *Nipponbare* in 2008. The homozygous plants of *myb103* were grown in the experimental field of Huazhong Agricultural University from 2011 to 2019 (Wuhan, China). To identify the gene of *Osfc9* mutant, a F_2_ mapping population was generated by crossing *Osfc9* with MH63 (a wild-type polymorphic *indica* variety). Map-based cloning approach was used for gene identification of *Osfc9*, based on 1050 F_2_ mutant plants with public SSR (Simple Sequence Repeats) molecular markers. Using 145 polymorphic markers, the *Osfc9* gene was preliminarily localized between markers RM22342 and RM22392 on chromosome 8 (Fig. 1). We further used markers (RM5647, RM22358, RM4955) and primers SSR1–SSR5 (Additional file 2: Table S1) to finally map the gene between SSR2 and RM4955 in a 46-kb genomic region, which contained four open reading frames. These four genes were cloned by amplifying the CDS sequences using KOD-Plus (TOYOBO, Japan), and only one gene, *LOC_Os08g05520* named *OsMYB103L*, was found with 18-base deletion.

For genetic complementation of *Osfc9* mutant, a 2851-bp full genomic sequence of *LOC_Os08g05520* driven by its upstream 2 kb sequence as promoter was cloned into the binary vector pC1300T. This complementary vector was introduced into *Agrobacterium tumefaciens* strain *EHA105* and transformed into the *Osfc9* mutant. Positive T_0_ transgenic lines were selected using hygromycin (Hyg) as plant selection marker.

### Generation of RNAi-silenced *OsMYB103L* transgenic lines

According to the full-length CDS of *OsMYB103L*, the 395-bp 3’ fragment was cloned into pTCK303 vector using RNAi-F and RNAi-R primers (Additional file 2: Table S1). The RNA interference (RNAi) vector of *OsMYB103L* was introduced into *Agrobacterium tumefaciens* strain *EHA105* and transformed into *Nipponbare* rice. Positive T_0_ transgenic lines were selected using Hyg as plant selection marker, and segregating T_1_ plants were screened by PCR and verified by quantitative real time-PCR (qRT-PCR).

### Total RNA isolation and qRT‑PCR analysis

Rice samples were collected from second internodes of stem tissues at heading stage. Total RNAs were extracted using Trizol reagent (Invitrogen) and reverse-transcribed into cDNA with the GoScript ™ Reverse Transcription System (Promega). qRT-PCR was independently performed by using the SYBR Green PCR Master Mixture (ZF101, ZOMANBIO) with three biological replicates as described [46].

### Measurements of stem strength and agronomic traits

Rice stem strength and lodging index were measured as described previously [25, 29]. The breaking resistance of rice third internode was detected using a prostrate tester (DIK7401, Japan) with the distance between fulcra of the tester at 5 cm. Fresh weight (W) of the upper portion of the rice plant was measured including panicle and the three internodes, leaf and leaf sheath. Bending moment (BM) and lodging index (LI) were, respectively, calculated using the following formulae: BM = length from the third internode to the top of panicle × W; and LI = BM/breaking resistance. In addition, 1000-grain weight was weighed after the mature rice samples were dried in the oven at 60 °C. All measurements were conducted under ten independent biological duplicates.

### GUS staining analysis

The promoter of *OsMYB103L* (about 2.2 kb upstream of ATG) was amplified from the rice genomic DNA (*Nipponbare*) using the primers GUS-F and GUS-R (Additional file 6: Table S1). The DNA fragment was then cloned into the pC1301gT vector resulting in fusion of the promoter and the *β-glucuronidase* reporter gene. The pro*OsMYB103L*: GUS construct was transformed into wild type (WT) rice cultivar *Nipponbare*. Three independent transgenic lines were obtained after screening using Hyg marker. GUS staining of stem tissue was performed as previously described [47].

### Subcellular localization and transactivation assay

For observation of *OsMYB103L* subcellular localization, the full-length *OsMYB103L* sequence was cloned into pC1300-EGFP to create 35S: *OsMYB103L*-EGFP vector using primers (EGFP-F and EGFP-R) listed in Additional file 2: Table S1. The construct was transformed into *Agrobacterium* strain *GV3101* and infiltrated into tobacco leaves for confocal microscopy. The subcellular localization of fused enhanced green fluorescent protein (EGFP) was visualized using a confocal laser scanning microscope (Leica SP5 CLSM) as previously described [48].

A yeast strain (*AH109*) containing HIS3 and ADE2 reporter genes was used to analyze transactivation of *OsMYB103L* as previously described [17]. The full-length coding sequence of *OsMYB103L* was amplified using BD-F and BD-R primers (Additional file 6: Table S1), cloned into pGBKT7 by fusion with the GAL4 DNA-binding domain, and then transformed into the *AH109*. The empty pGBKT7 (BD) was used as negative control. The transactivation activity of these proteins was evaluated according to the growth on SD/-Trp, -His, -Ade (synthetic defined, tryptophan, histidine, adenine dropout).

### Observation of plant cell walls and cellulose microfibrils

Cellulose staining by S4B was performed as previously described with little modifications [49]. The rice second internodes of stem (0.5 cm sections above the node) at the heading stage were sectioned with 100 μm thickness using a microtome (VT1000S, Leica). The sections were stained with 0.01% S4B in 50 mM NaCl for 15 min, and washed three times using phosphate buffered saline (PBS) buffer. The stained sections were observed using a Nikon Eclipse Ni-U microscope under 530–560 nm excitation and 620–660 nm emission and bright field to analyze cellulose distribution and cell morphology.

TEM was used to observe cell wall structures in the middle 0.5-cm sections from the third leaf veins of three-leaf-old rice plants as previously described [25, 29]. The samples were post-fixed in 2% (w/v) OsO_4_ for 1 h after extensively washing in the PBS buffer and embedded with Super Kit (Sigma), cut with an Ultracut E ultramicrotome (Leica) and picked up on Formvar-coated copper grids. After post-staining with uranyl acetate and lead citrate, the specimens were viewed under a Hitachi H7650 (Hitachi Ltd., Tokyo, Japan) transmission electron microscope.

AFM was applied to observe cellulose microfibrils as described previously [32]. The basal region of the second internodes of rice plants at heading stage were sectioned with 100 μm thickness, and then incubated with 8% acidic chlorite (1 mM HCl added to 1 g sodium chlorite) at 50 °C for 48 h. After washed with distilled water five times, the sections were used for AFM (CSPM5500) observation.

### Detection of cellulose features (DP, CrI)

The DP and CrI of cellulose substrates were, respectively, detected as previously described [24, 25, 50, 51]. For cellulose DP assay, the crude cellulose samples were prepared by extracting raw biomass materials with 4 M KOH (containing sodium borohydride at 1.0 mg mL^−1^) and 8% (w/v) NaClO_2_, and all experiments were conducted in independent triplicate. For cellulose CrI assay, the X-ray diffraction method was performed using a Rigaku D/MAX instrument (Ultima III, Japan). Technical standard errors of the CrI method were measured as ± 0.05–0.15 using five representative samples in independent triplicate.

### Fourier transform infrared spectroscopy scanning

A PerkinElmer spectrophotometer (NEXUS 470, Thermo Fisher Scientific, Waltham, MA, USA) was applied to scan biomass samples and the he Fourier transform infrared (FTIR) spectra were recorded in absorption mode over 32 scans at a resolution of 4 cm^−1^ in the range of 4000 to 400 cm^−1^ as previously described [24, 50].

### Plant cell wall fractionation and determination

Plant cell wall fractionation procedure was applied to extract wall polymers as previously described [24, 29]. The well-mixed biomass powders were ground with potassium phosphate buffer (pH 7.0), followed with chloroform–methanol (1:1, v/v), DMSO–water (9:1, v/v) and 0.5% (w/v) ammonium oxalate to consequently remove soluble sugars and protein, lipid, starch and pectin. The remaining residue was extracted with 4 M KOH containing 1.0 mg mL^−1^ sodium borohydride, yielding KOH-extractable hemicelluloses. The residue was finally added with H_2_SO_4_ (67%, v/v) to completely dissolve cellulose and non-KOH-extractable hemicelluloses. Cellulose content was calculated by determining the hexoses using the anthrone/H_2_SO_4_ method. Total hemicelluloses were calculated by determining the hexoses and pentoses using the orcinol/HCl method. Lignin content was measured by the two-step acid hydrolysis method according to the NREL’s laboratory analytical protocol (LAP). All experimental analyses were completed in independent triplicate.

### Biomass pretreatment and enzymatic hydrolysis

Three biomass pretreatments and sequential enzymatic hydrolysis were performed as described previously [24, 26, 33] with minor modifications. For acid pretreatment, the well-mixed biomass powder (0.300 g) was added to 6 mL 1% H_2_SO_4_ (v/v), heated at 121 °C for 20 min in an autoclave (0.15 MPa), and then shaken under 150 rpm at 50 °C for 2 h. For alkali pretreatment, the biomass power was added into 6 mL 1% NaOH (w/v), and incubated at 50 °C under 150 rpm shaking for 2 h. For liquid hot water pretreatment, the well-mixed biomass powder was added into well-sealed stainless steel capsules at 12.5% solid loading, and heated at 200 °C under 15 rpm shaking for 30 min.

The pretreated biomass residues were washed with distilled water five times, and then washed once with 10 mL of mixed-cellulase reaction buffer. The washed residues were incubated with 6 mL (2 g L^−1^) of mixed cellulases (containing cellulases at 10.60 FPU g^−1^ biomass and xylanase at 6.72 U g^−1^ biomass from Imperial Jade Bio-technology Co., Ltd) at 5% solid loading, and shaken under 150 rpm at 50 °C for 48 h, while 1% Tween-80 was co-supplied. After the samples were centrifuged at 3000 g for 5 min, the supernatants were collected for hexoses and pentose assay. All experiments were carried out in independent triplicate.

### DAP-seq assay

The DNA affinity purification sequencing (DAP-seq) experiments and data analysis were conducted as previously described with minor modification [35]. For experiments, DNA was first extracted from young leaf tissue of rice cultivar *Nipponbare*, and fragmented by Bioruptor Plus to 250 bp. After selecting targeted fragments by magnetic beads (KAPA Pure Beads, KK8002), the fragmented DNA was successively performed with end repair, A-tailing and adapter ligation steps. The ampDAP-Seq DNA library was prepared by PCR amplification using KAPA HiFi HotStart ReadyMix with unique index primers, and selectively recovered as different DNA libraries. The full-lengths of *OsMYB103L* and *Osfc9* were, respectively, cloned to the HALO-tagged vector (pFN19K HaloTag^®^ T7 SP6 Flexi^®^ Vector) using DAP-F and DAP-R primers (Additional file 2: Table S1). HALO-tagged OsMYB103L and OsFC9 were expressed in vitro in a wheat germ system, and HALO-tagged GFP was expressed as control. The protein expression was detected by Western blot using HALO-Tag antibody. HALO-OsMYB103L and OsFC9 were immobilized on Magne HALO-Tag beads, and incubated with the DNA library. After bead washing, DNA was eluted and amplified with corresponding indexed primers. The final products were selected from 250 to 500 bp, and sent to ANOROAD company for ChIP-seq sequencing.

For DAP-seq analysis, sequencing fastq files were trimmed using trimmomatic (v0.32) [52]. Trimmed reads were mapped to the *Oryza sativa* IRGSP reference genome using bowtie2 (v2.2.2) [53]. The mapped reads were filtered for unique mapping reads and reads containing > MAPQ30 using samtools (v0.1.13) [54] to restrict reads aligned to multiple positions in the genome. MAPQ-filtered reads were used for peak calling using macs2 (v2.1.1) [55]. The predicted motif fasta sequence files were generated by extracting 50 bp upstream and downstream of the peak summit. All motifs were determined using meme-chip (v5.0.5) [56]. The target gene identification was defined as the closest gene containing a peak within 1 kb upstream and downstream and got by getfasta utilities of bedtools (v2.25.0) [57].

### RNA-seq assay

Total RNA was isolated using the Trizol Reagent (Invitrogen Life Technologies). Sequencing libraries were generated using the TruSeq RNA Sample Preparation Kit (Illumina, San Diego, CA, USA). mRNA was purified from total RNA using poly-T oligo-attached magnetic beads. Fragmentation was carried out using divalent cations under elevated temperature in an Illumina proprietary fragmentation buffer. First strand cDNA was synthesized using random oligonucleotides and SuperScript II, and second strand cDNA synthesis was subsequently performed using DNA Polymerase I and RNase H. Remaining overhangs were converted into blunt ends via exonuclease/polymerase activities and the enzymes were removed. After adenylation of the 3’ ends of the DNA fragments, Illumina PE adapter oligonucleotides were ligated to prepare for hybridization. DNA fragments with ligated adaptor molecules on both ends were selectively enriched using Illumina PCR Primer Cocktail in a 15 cycle PCR reaction. Products were purified (AMPure XP system) and quantified using the Agilent high sensitivity DNA assay on a Bioanalyzer 2100 system (Agilent). The sequencing library was then sequenced on a Hiseq platform (Illumina) by Shanghai Personal Biotechnology Cp. Ltd. For data analysis, genes with fold change ≥ 2.0 and significance level *p* ≤ 0.05 were differentially expressed. DEGs were annotated against the GO (Gene Ontology). Each sample (mutant, wild type) was analyzed in biological triplicate.

### Gene co-expression analysis and other bioinformatics

Gene co-expression analysis has been conducted as previously described with modifications [58]. The Affymetrix Rice GeneChip Genome Array microarray datasets of candidate genes were obtained from CREP (Collections of Rice Expression Profiling, http://crep.ncpgr.cn) from an *indica* variety (Zhenshan 97).

The predictions of the three-dimensional structure of OsMYB103L and mutational protein OsFC9 were conducted using the SWISS-MODEL (https://swissmodel.expasy.org/). The volcano plot was drawn by GraphPad Prism 9.0.0. The vein plot was online completed by DeepVein (http://www.deepvenn.com/). GO analysis was performed using online agriGO database v2.0 [38]. The gene co-expression heatmap was constructed by using pheatmap of R documentation.

### Data statistical analysis

The SPSS statistical software was used for data analysis. Student’s t test was used for comparison analysis. Significance was measured at the levels of *p* < 0.05 and *p* < 0.01. All experiments were conducted with at least three independent replicates.

## Supplementary Information

**Additional file 1: Figure S1.** Analysis of agronomic traits of *myb103* mutant and *OsMYB103L*-RNAi transgenic plant. **A** Stem strength, **B** plant height, **C** lodging index and **D** 1000-Grain weight of *myb103* mutant and *OsMYB103L*-RNAi transgenic plant (RNAi-1) compared with WT. **As significant differences between *myb103* and RNAi-1 with WT at *p* < 0.01 (*n* = 10). **Figure S2**. Quantitative measurement of cell wall thickness by transmission electron microscopy in *myb103* mutant and *OsMYB103L*-RNAi transgenic plant. * and ** As significant differences between *myb103* and RNAi-1 with WT at *p* < 0.05 and 0.01 (n=3), respectively. **Figure S3.** Fourier transform infrared spectroscopic profiling of mature straws of the *myb103* mutant and *OsMYB103L*-RNAi-1 transgenic line. **Figure S4.** Peak enrichment of *OsCESA4*, *OsCESA7* and *OsCESA9* by *OsMYB103L* through DAP-seq analysis. **Figure S5.** Subcellular localization and transactivation analysis of *OsMYB103L*. **A** Subcellular localization of *OsMYB103L* in tobacco. **B** Transcription activation analysis of OsMYB103L in yeast.

**Additional file 2: Table S1.** Primers used in this study.

**Additional file 3: Table S2.** Target genes of *OsMYB103L* by using DAP-seq analysis.

**Additional file 4: Table S3.** Genes that up- or down-regulated by twofold in DGE data of *myb103* mutant compared to that of the wild type.

**Additional file 5: Table S4.** 149 overlapped genes related to *OsMYB103L* regulation by using multi-omics analysis.

**Additional file 6: Table S5.** Analysis of AC elements and SMREs in the promoters of candidate genes regulated by *OsMYB103L*.

## Data Availability

The datasets (including the RNA-seq of *Osfc9* mutant and WT, the DAP-seq of OsMYB103L and OsFC9) were submitted to the SRA (Sequence Read Archive) repository (PRJNA743986). All data generated or analyzed during this study are included in this published article and its additional files.

## Abbreviations

AFM: Atomic force microscopy
CrI: Crystalline index
DAP-seq: DNA affinity purification sequencing
DP: Degree of polymerization
FTIR: Fourier transform infrared
GO: Gene Ontology
GUS: β-Glucuronidase
RNAi: RNA interference
S4B: Pontamine fast scarlet 4B
TEM: Transmission electron microscopy

## References

[1] Wang Y, Liu P, Zhang G, Yang Q, Lu J, Xia T, Peng L, Wang Y (2021). Cascading of engineered bioenergy plants and fungi sustainable for low-cost bioethanol and high-value biomaterials under green-like biomass processing. Renew Sustain Energy Rev.

[2] Seddiqi H, Oliaei E, Honarkar H, Jin J, Geonzon LC, Bacabac RG, Klein-Nulend J (2021). Cellulose and its derivatives: towards biomedical applications. Cellulose.

[3] Peng L, Kawagoe Y, Hogan P, Delmer D (2002). Sitosterol-β-glucoside as primer for cellulose synthesis in plants. Science.

[4] Somerville C (2006). Cellulose synthesis in higher plants. Annu Rev Cell Dev Biol.

[5] McFarlane HE, Doring A, Persson S (2014). The cell biology of cellulose synthesis. Annu Rev Plant Biol.

[6] Polko JK, Kieber JJ (2019). The regulation of cellulose biosynthesis in plants. Plant Cell.

[7] Li S, Bashline L, Lei L, Gu Y (2014). Cellulose synthesis and its regulation. Arabidopsis Book..

[8] Persson S, Paredez A, Carroll A, Palsdottir H, Doblin M, Poindexter P, Khitrov N, Auer M, Somerville CR (2007). Genetic evidence for three unique components in primary cell-wall cellulose synthase complexes in *Arabidopsis*. Proc Natl Acad Sci U S A.

[9] Hu H, Zhang R, Feng S, Wang Y, Wang Y, Fan C, Li Y, Liu Z, Schneider R, Xia T, Ding S, Persson S, Peng L (2018). Three AtCesA6-like members enhance biomass production by distinctively promoting cell growth in *Arabidopsis*. Plant Biotechnology J.

[10] Zhang B, Gao Y, Zhang L, Zhou Y (2021). The plant cell wall: biosynthesis, construction, and functions. J Integr Plant Biol.

[11] Nakano Y, Yamaguchi M, Endo H, Rejab NA, Ohtani M (2015). NAC-MYB-based transcriptional regulation of secondary cell wall biosynthesis in land plants. Front Plant Sci.

[12] Zhong R, Lee C, Ye ZH (2010). Evolutionary conservation of the transcriptional network regulating secondary cell wall biosynthesis. Trends Plant Sci.

[13] Zhong R, Ye ZH (2012). MYB46 and MYB83 bind to the SMRE sites and directly activate a suite of transcription factors and secondary wall biosynthetic genes. Plant Cell Physiol.

[14] Zhou J, Lee C, Zhong R, Ye ZH (2009). MYB58 and MYB63 are transcriptional activators of the lignin biosynthetic pathway during secondary cell wall formation in *Arabidopsis*. Plant Cell.

[15] Ohman D, Demedts B, Kumar M, Gerber L, Gorzsas A, Goeminne G, Hedenstrom M, Ellis B, Boerjan W, Sundberg B (2013). MYB103 is required for *FERULATE-5-HYDROXYLASE* expression and syringyl lignin biosynthesis in *Arabidopsis* stems. Plant J.

[16] Yang C, Li D, Liu X, Ji C, Hao L, Zhao X, Li X, Chen C, Cheng Z, Zhu L (2014). OsMYB103L, an R2R3-MYB transcription factor, influences leaf rolling and mechanical strength in rice (*Oryza sativa* L.). BMC Plant Biol.

[17] Ye Y, Liu B, Zhao M, Wu K, Cheng W, Chen X, Liu Q, Liu Z, Fu X, Wu Y (2015). CEF1/OsMYB103L is involved in GA-mediated regulation of secondary wall biosynthesis in rice. Plant Mol Biol.

[18] Darley CP, Forrester AM, McQueen-Mason SJ (2001). The molecular basis of plant cell wall extension. Plant Mol Biol.

[19] Taylor NG (2008). Cellulose biosynthesis and deposition in higher plants. New Phytol.

[20] Ding SY, Liu YS, Zeng Y, Himmel ME, Baker JO, Bayer EA (2012). How does plant cell wall nanoscale architecture correlate with enzymatic digestibility?. Science.

[21] Li M, Feng S, Wu L, Li Y, Fan C, Zhang R, Zou W, Tu Y, Jing HC, Li S, Peng L (2014). Sugar-rich sweet sorghum is distinctively affected by wall polymer features for biomass digestibility and ethanol fermentation in bagasse. Bioresour Technol.

[22] Zhang W, Yi Z, Huang J, Li F, Hao B, Li M, Hong S, Lv Y, Sun W, Ragauskas A, Hu F, Peng J, Peng L (2013). Three lignocellulose features that distinctively affect biomass enzymatic digestibility under NaOH and H_2_SO_4_ pretreatments in *Miscanthus*. Bioresour Technol.

[23] Wu Z, Zhang M, Wang L, Tu Y, Zhang J, Xie G, Zou W, Li F, Guo K, Li Q, Gao C, Peng L (2013). Biomass digestibility is predominantly affected by three factors of wall polymer features distinctive in wheat accessions and rice mutants. Biotechnol Biofuels.

[24] Wu L, Feng S, Deng J, Yu B, Wang Y, He B, Peng H, Li Q, Hu R, Peng L (2019). Altered carbon assimilation and cellulose accessibility to maximize bioethanol yield under low-cost biomass processing in corn brittle stalk. Green Chem.

[25] Li F, Xie G, Huang J, Zhang R, Li Y, Zhang M, Wang Y, Li A, Li X, Xia T, Qu C, Hu F, Ragauskas A, Peng L (2017). OsCESA9 conserved-site mutation leads to largely enhanced plant lodging resistance and biomass enzymatic saccharification by reducing cellulose DP and crystallinity in rice. Plant Biotechnol J.

[26] Li Y, Liu P, Huang J, Zhang R, Hu Z, Feng S, Wang Y, Wang L, Xia T, Peng L (2018). Mild chemical pretreatments are sufficient for bioethanol production in transgenic rice straws overproducing glucosidase. Green Chem.

[27] Huang J, Xia T, Li G, Li X, Li Y, Wang Y, Wang Y, Chen Y, Xie G, Bai FW, Peng L, Wang L (2019). Overproduction of native endo-β-1,4-glucanases leads to largely enhanced biomass saccharification and bioethanol production by specific modification of cellulose features in transgenic rice. Biotechnol Biofuels.

[28] Domínguez-Escribá L, Porcar M (2010). Rice straw management: the big waste. Biofuels Bioprod Biorefin.

[29] Fan C, Feng S, Huang J, Wang Y, Wu L, Li X, Wang L, Tu Y, Xia T, Li J, Cai X, Peng L (2017). AtCesA8-driven *OsSUS3* expression leads to largely enhanced biomass saccharification and lodging resistance by distinctively altering lignocellulose features in rice. Biotechnol Biofuels.

[30] Goshadrou A, Lefsrud M (2017). Synergistic surfactant-assisted [EMIM]OAc pretreatment of lignocellulosic waste for enhanced cellulose accessibility to cellulase. Carbohydr Polym.

[31] Wang Q, Wei W, Kingori GP, Sun J (2015). Cell wall disruption in low temperature NaOH/urea solution and its potential application in lignocellulose pretreatment. Cellulose.

[32] Zhang R, Hu H, Wang Y, Hu Z, Ren S, Li J, He B, Wang Y, Xia T, Chen P, Xie G, Peng L (2020). A novel rice *fragile culm 24* mutant encodes a UDP-glucose epimerase that affects cell wall properties and photosynthesis. J Exp Bot.

[33] Hu M, Yu H, Li Y, Li A, Cai Q, Liu P, Tu Y, Wang Y, Hu R, Hao B, Peng L, Xia T (2018). Distinct polymer extraction and cellulose DP reduction for complete cellulose hydrolysis under mild chemical pretreatments in sugarcane. Carbohydr Polym.

[34] O’Malley RC, Huang SSC, Song L, Lewsey MG, Bartlett A, Nery JR, Galli M, Gallavotti A, Ecker JR (2016). Cistrome and epicistrome features shape the regulatory DNA landscape. Cell.

[35] Bartlett A, O’Malley RC, Huang S-sC, Galli M, Nery JR, Gallavotti A, Ecker JR (2017). Mapping genome-wide transcription-factor binding sites using DAP-seq. Nat Protoc.

[36] Bienert S, Waterhouse A, de Beer Tjaart AP, Tauriello G, Studer G, Bordoli L, Schwede T (2017). The SWISS-MODEL Repository: new features and functionalities. Nucleic Acids Res.

[37] Kusanagi K, Kawabata M, Mishima HK, Miyazono K (2001). α-Helix 2 in the amino-terminal Mad Homology 1 Domain is responsible for specific DNA binding of Smad3. J Biol Chem.

[38] Tian T, Liu Y, Yan H, You Q, Yi X, Du Z, Xu W, Su Z (2017). agriGO v2.0: a GO analysis toolkit for the agricultural community, 2017 update. Nucleic Acids Res.

[39] Xie G, Peng L (2011). Genetic engineering of energy crops: a strategy for biofuel production in China. J Integr Plant Biol.

[40] Zhang B, Zhou Y (2011). Rice brittleness mutants: a way to open the 'black box' of monocot cell wall biosynthesis. J Integr Plant Biol.

[41] Wu L, Han L, Li Q, Wang G, Zhang H, Li L (2021). Using Interactome Big Data to crack genetic mysteries and enhance future crop breeding. Mol Plant.

[42] Li Y, Qian Q, Zhou Y, Yan M, Sun L, Zhang M, Fu Z, Wang Y, Han B, Pang X, Chen M, Li J (2003). BRITTLE CULM1, which encodes a COBRA-like protein, affects the mechanical properties of rice plants. Plant Cell.

[43] Hirano K, Kotake T, Kamihara K, Tsuna K, Aohara T, Kaneko Y, Takatsuji H, Tsumuraya Y, Kawasaki S (2010). Rice BRITTLE CULM 3 (BC3) encodes a classical dynamin OsDRP2B essential for proper secondary cell wall synthesis. Planta.

[44] Xiong G, Li R, Qian Q, Song X, Liu X, Yu Y, Zeng D, Wan J, Li J, Zhou Y (2010). The rice dynamin-related protein DRP2B mediates membrane trafficking, and thereby plays a critical role in secondary cell wall cellulose biosynthesis. Plant J.

[45] Wang Y, Fan C, Hu H, Li Y, Sun D, Wang Y, Peng L (2016). Genetic modification of plant cell walls to enhance biomass yield and biofuel production in bioenergy crops. Biotechnol Adv.

[46] Fan C, Wang G, Wu L, Liu P, Huang J, Jin X, Zhang G, He Y, Peng L, Luo K, Feng S (2020). Distinct cellulose and callose accumulation for enhanced bioethanol production and biotic stress resistance in OsSUS3 transgenic rice. Carbohydr Polym.

[47] Jefferson RA, Kavanagh TA, Bevan MW (1987). GUS fusions: beta-glucuronidase as a sensitive and versatile gene fusion marker in higher plants. EMBO J.

[48] Wang Y, Li D, Gao J, Li X, Zhang R, Jin X, Hu Z, Zheng B, Persson S, Chen P (2017). The 2'-O-methyladenosine nucleoside modification gene *OsTRM13* positively regulates salt stress tolerance in rice. J Exp Bot.

[49] Yang B, Voiniciuc C, Fu L, Dieluweit S, Klose H, Usadel B (2019). TRM4 is essential for cellulose deposition in *Arabidopsis* seed mucilage by maintaining cortical microtubule organization and interacting with CESA3. New Phytol.

[50] Gao H, Wang Y, Yang Q, Peng H, Li Y, Zhan D, Wei H, Lu H, Bakr MMA, Ei-Sheekh MM, Qi Z, Peng L, Lin X (2021). Combined steam explosion and optimized green-liquor pretreatments are effective for complete saccharification to maximize bioethanol production by reducing lignocellulose recalcitrance in one-year-old bamboo. Renew Energy.

[51] Hu Z, Wang Y, Liu J, Li Y, Wang Y, Huang J, Ai Y, Chen P, He Y, Aftab MN, Wang L, Peng L (2021). Integrated NIRS and QTL assays reveal minor mannose and galactose as contrast lignocellulose factors for biomass enzymatic saccharification in rice. Biotechnol Biofuels.

[52] Bolger AM, Lohse M, Usadel B (2014). Trimmomatic: a flexible trimmer for Illumina sequence data. Bioinf.

[53] Langmead B, Salzberg SL (2012). Fast gapped-read alignment with Bowtie 2. Nat Methods.

[54] Li H, Handsaker B, Wysoker A, Fennell T, Ruan J, Homer N, Marth G, Abecasis G, Durbin R (2009). Genome Project Data P. The Sequence Alignment/Map format and SAMtools. Bioinf.

[55] Zhang Y, Liu T, Meyer CA, Eeckhoute J, Johnson DS, Bernstein BE, Nussbaum C, Myers RM, Brown M, Li W, Liu XS (2008). Model-based analysis of ChIP-Seq (MACS). Genome Biol.

[56] Machanick P, Bailey TL (2011). MEME-ChIP: motif analysis of large DNA datasets. Bioinf.

[57] Quinlan AR, Hall IM (2010). BEDTools: a flexible suite of utilities for comparing genomic features. Bioinf.

[58] Guo K, Zou W, Feng Y, Zhang M, Zhang J, Tu F, Xie G, Wang L, Wang Y, Klie S, Persson S, Peng L (2014). An integrated genomic and metabolomic framework for cell wall biology in rice. BMC Genomics.

