# Supplementary figures and images for: Down-regulation of OsMYB103L distinctively alters beta-1,4-glucan polymerization and cellulose microfibers assembly for enhanced biomass enzymatic saccharification in rice
Source: Biotechnol Biofuels. 2021 Dec 27;14:245. doi: 10.1186/s13068-021-02093-8 (PMC8713402; doi:10.1186/s13068-021-02093-8)

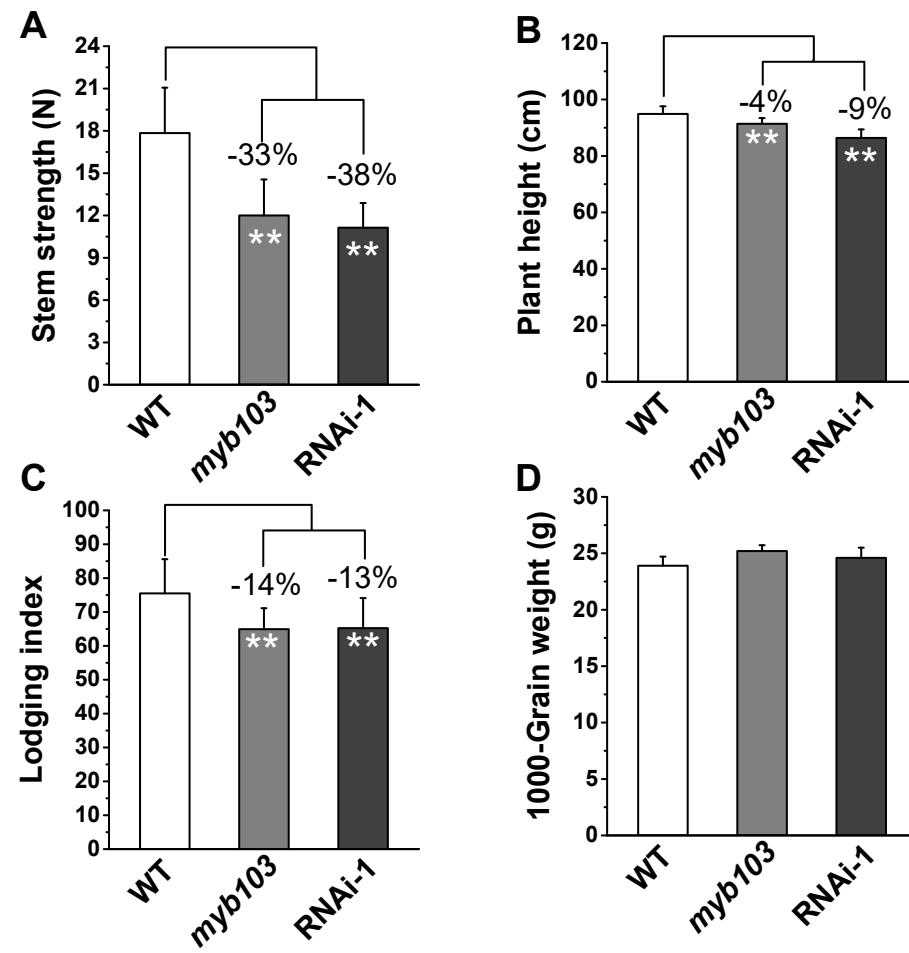

Figure S1

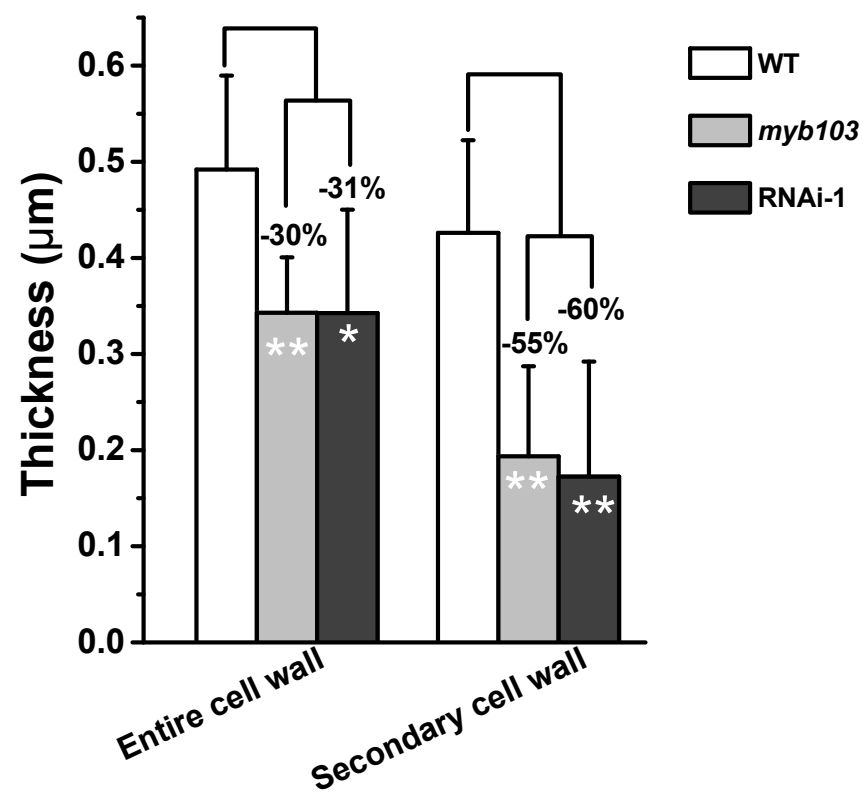

Figure S2

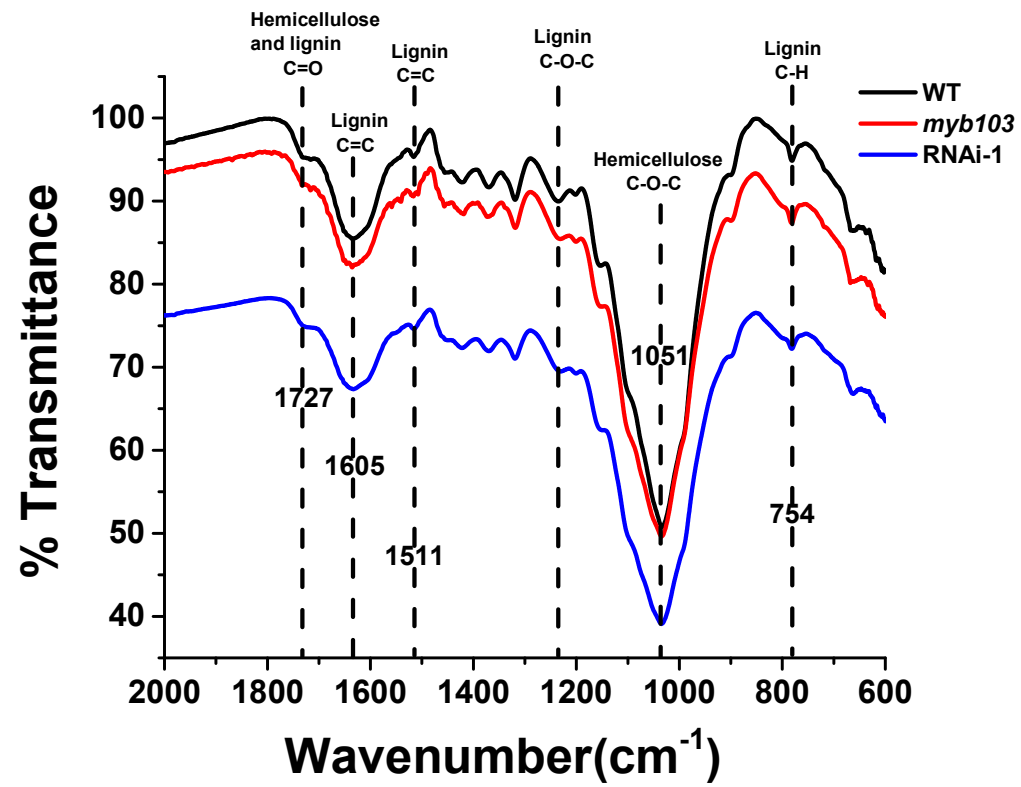

Figure S3

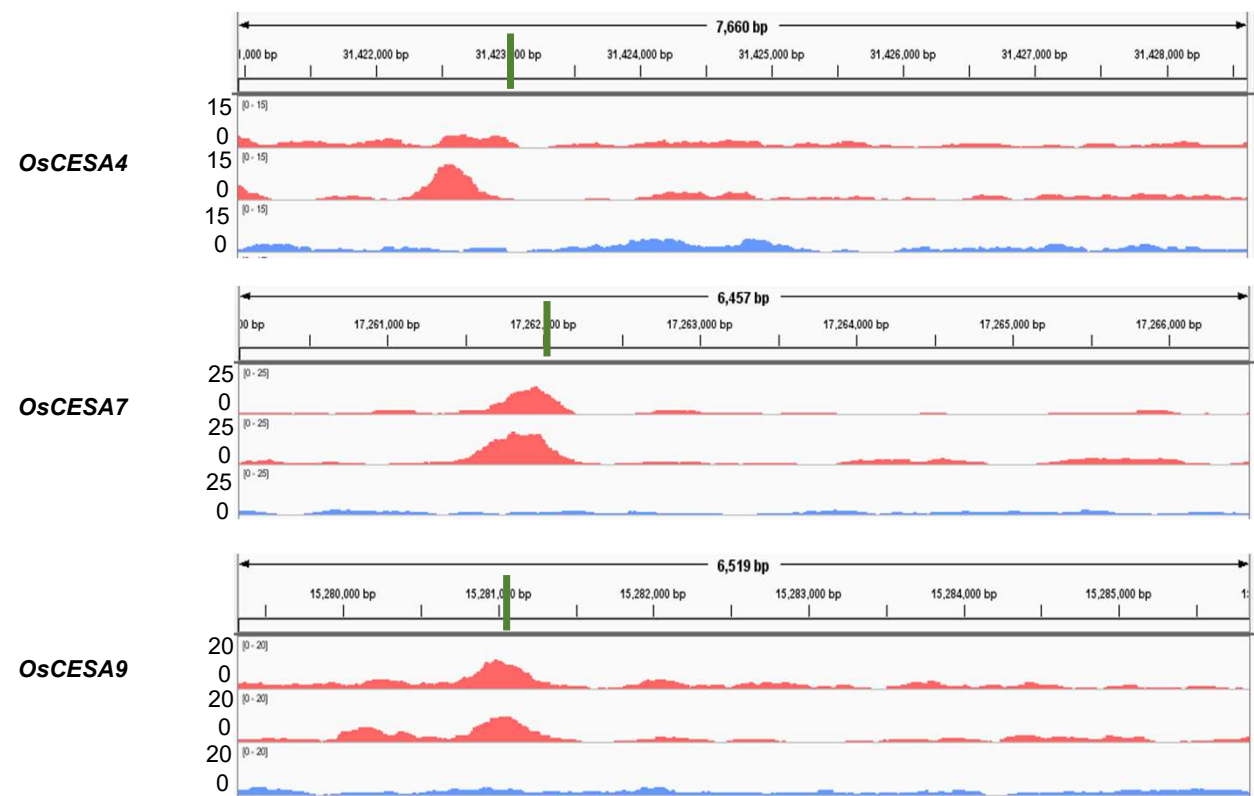

**Figure S4**

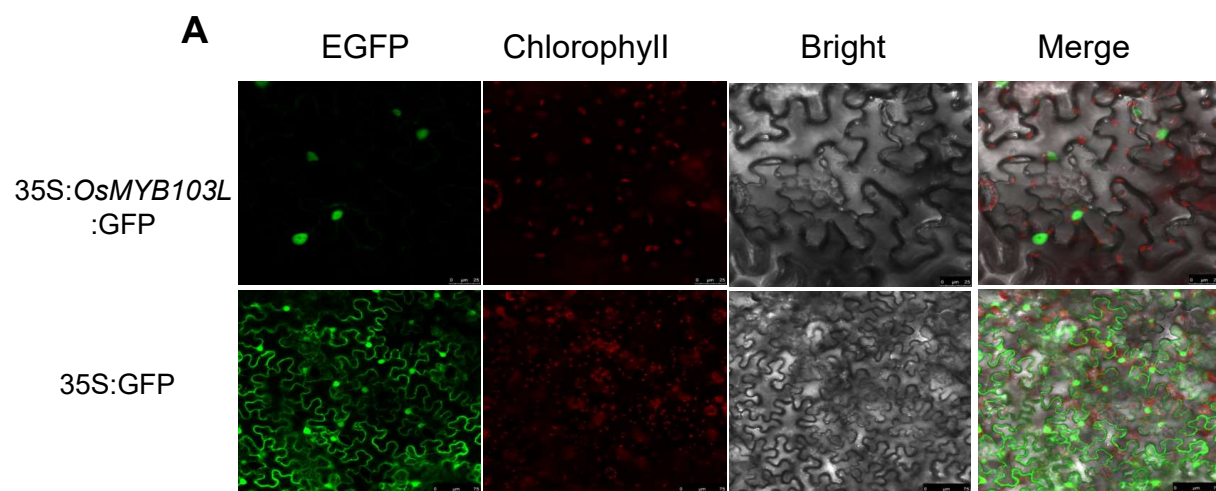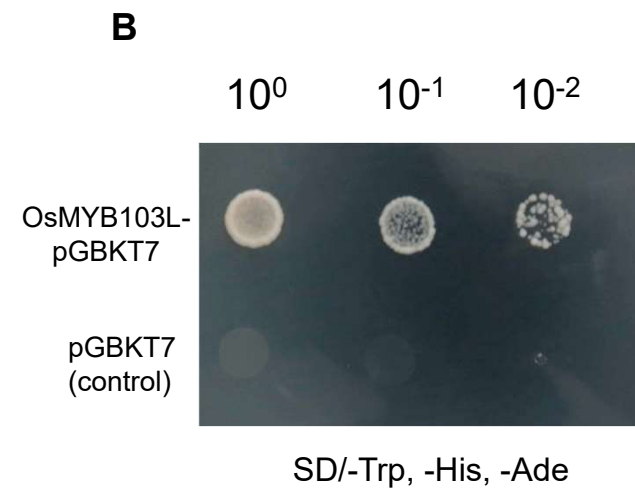

**Figure S5**

Supplement: Supplementary file 1 — Additional file 1: Figure S1. Analysis of agronomic traits of myb103 mutant and OsMYB103L-RNAi transgenic plant. A Stem strength, B plant height, C lodging index and D 1000-Grain weight of myb103 mutant and OsMYB103L-RNAi transgenic plant (RNAi-1) compared with WT. **As significant differences between myb103 and RNAi-1 with WT at p < 0.01 (n = 10). Figure S2. Quantitative measurement of cell wall thickness by transmission electron microscopy in myb103 mutant and OsMYB103L-RNAi transgenic plant. * and ** As significant differences between myb103 and RNAi-1 with WT at p < 0.05 and 0.01 (n=3), respectively. Figure S3. Fourier transform infrared spectroscopic profiling of mature straws of the myb103 mutant and OsMYB103L-RNAi-1 transgenic line. Figure S4. Peak enrichment of OsCESA4, OsCESA7 and OsCESA9 by OsMYB103L through DAP-seq analysis. Figure S5. Subcellular localization and transactivation analysis of OsMYB103L. A Subcellular localization of OsMYB103L in tobacco. B Transcription activation analysis of OsMYB103L in yeast. [file 13068_2021_2093_MOESM1_ESM.pdf]
